# NMR Structure and CD Titration with Metal Cations of Human Prion α2-Helix-Related Peptides

**DOI:** 10.1155/2007/10720

**Published:** 2007-09-27

**Authors:** Luisa Ronga, Pasquale Palladino, Gabriella Saviano, Teodorico Tancredi, Ettore Benedetti, Raffaele Ragone, Filomena Rossi

**Affiliations:** ^1^Dipartimento delle Scienze Biologiche, Centro Interuniversitario di Ricerca sui Peptidi Bioattivi, Università Federico II di Napoli e Istituto di Biostrutture e Bioimmagini, CNR, Via Mezzocannone 16, 80134 Napoli, Italy; ^2^Dipartimento di Scienze e Tecnologie per l'Ambiente e il Territorio, Università del Molise, Contrada Fonte Lappone, 86090 Pesche, Italy; ^3^Istituto di Chimica Biomolecolare, CNR, Via Campi Flegrei 34, 80078 Pozzuoli, Italy; ^4^Dipartimento di Biochimica e Biofisica e CRISCEB, Seconda Università di Napoli, Via Santa Maria di Costantinopoli 16, 80138 Napoli, Italy

## Abstract

The 173–195 segment corresponding to the helix 2 of the C-globular prion protein domain could be one of several “spots” of intrinsic conformational flexibility. In fact, it possesses chameleon conformational behaviour and gathers several disease-associated point mutations. We have performed spectroscopic studies on the wild-type fragment 173–195 and on its D178N mutant dissolved in trifluoroethanol to mimic the in vivo system, both in the presence and in the absence of metal cations. NMR data showed that the structure of the D178N mutant is characterized by two short helices separated by a kink, whereas the wild-type peptide is fully helical. Both peptides retained these structural organizations, as monitored by CD, in the presence of metal cations. NMR spectra were however not in favour of the formation of definite ion-peptide complexes. This agrees with previous evidence that other regions of the prion protein are likely the natural target of metal cation binding.

## 1. INTRODUCTION

The cellular prion protein is a synaptic glycoprotein expressed in the central nervous system, in lymphatic tissue, and at neuromuscular junctions [1]. It is abundantly spread in the brain of mammals, where it is attached to the cell membrane by a glycosylphosphatidylinositol anchor [2]. Although its physiological function is still largely unknown, PrP protein is unequivocally associated to the onset of a family of diseases named transmissible spongiform encephalopathies (TSE) [3] by a mechanism involving the conversion of the cellular form, ${\text{PrP}}^{\text{C}}$, into an insoluble (scrapie) variant, ${\text{PrP}}^{\text{Sc}}$, which is deemed to also retain an intrinsic infectivity [4]. These two PrP isomers substantially differ in their secondary structures [5–7]. Indeed, ${\text{PrP}}^{\text{C}}$ is predominantly *α*-helical with little *β*-sheet contribution [8, 9], whereas ${\text{PrP}}^{\text{Sc}}$ possesses a considerably higher *β*-sheet content, which suggests that isomerization is driven by a major misfolding event leading to more extensive *β*-sheet conformation.

According to this model, prion diseases are caused by a rearrangement of the cellular form into proteinase-K-resistant amyloidogenic, *β*-sheet-containing and potentially infective structural variants [10–12]. The role played by such variants in amyloid fibril formation and consequently in prion aetiopathogenesis is not definitely elucidated, nor has the molecular link between fibrils and disease yet been clearly established. Several experimental observations suggest that the interaction of PrP with membrane surface lipids [13, 14] or with a yet unknown protein X [15, 16] might play a pivotal role in the protein early transformation and conformational variability propagation. However, hypotheses currently under investigation clash with vagueness of information about ${\text{PrP}}^{\text{Sc}}$ structure. It has been also proposed that the amyloid-forming tendency in prions, as well as in other fibrillogenic proteins, could depend on the number of water exposed backbone H-bonds [17–19] and that tightly backbone-bound water molecules are of fundamental importance in local protein stability and folding. In fact, some protein regions exhibit an H-bond network poorly shielded from and more vulnerable to bulk water attack, thus being more prone to rearrangement. Such regions include specifically the helix 1, the C-terminal segment of the helix 2, the loop between helix 2 and helix 3 and some residues within the helix 3, which belong to the C-terminal globular domain. This domain has been implicated in the rearrangement mechanism and in the formation of toxic fibrils [20–25]. It has indeed been shown that ablation of any of the prion helices leads to protein variants unable to convert into ${\text{PrP}}^{\text{Sc}}$, while contextual removal of an N-terminal portion (residues 23–89) and of helix 1 (residues 144–157) produces highly infective prions [26, 27].

Particularly fascinating is the notion that the protein possesses one or several “spots” of intrinsic conformational weakness, which may lead the whole secondary and tertiary structure to succumb in favour of more stable, but aggregation-prone conformations, depending on pH, redox condition, or glycosylation [21, 28]. The C-terminal side of helix 2 is decidedly suspected to be one of such spots and, in this regard, has recently gained the attention of several investigations [25, 29–33]. From these studies, it emerges that the synthetic fragment corresponding to helix 2 is able to adopt either *α*-helix or *β*-sheet conformation, and that such a behaviour is likely under the control of the highly conserved threonine-rich stretch 188–195. Furthermore, the helix 2 fragment, also depending on the glycosylation state and the presence of metals [20–22], can be toxic to neuronal cells and strongly fibrillogenic, adding a further clue to the working hypothesis that it is involved in the protein aggregation process and in the toxicity associated to the scrapie variant.

Most recently, on the basis of H/D exchange data, Lu and coworkers [34] have mapped the H-bonded *β*-sheet core of PrP amyloid to the C-terminal region (starting at residue ≈169) that in the native structure of PrP monomer corresponds to *α*-helix 2, a major part of *α*-helix 3, and the loop between these two helices. As a matter of fact, several disease-causing point mutations are also gathered on this region, notably the D178N, V180I, T183A, H187R, T188R, T188K, T188A, which are presumed to induce further protein destabilization [31, 35–37] and to contribute to protein transformation.

The intriguing structural properties of this protein domain, as well as the influence that a disease-associated mutation can have on its relative stability, prompted us to perform comparative NMR and CD structural investigations on two peptides, hPrP[173–195] and hPrP[173–195] D178N. These are derived from the wild type and the Creutzfeldt-Jakob-disease-associated mutant [37] full length helix 2, respectively, and can be therefore considered as representative contributors to the conformational landscape of this region. Notably, these peptide fragments exhibited random organization in aqueous solution (Ronga et al., unpublished results). This is likely to be ascribed to the absence of mutual interactions with the other helical segments as well as of the interhelical disulphide bridge, which contribute to the integrity of the whole C-terminal globular domain in ${\text{PrP}}^{\text{C}}$. To avoid experimental ambiguity due to the fact that the parent segment in the native protein assumes helical conformation, we have mimicked native-like conditions using the *α*-inducer trifluoroethanol (TFE) to force both peptides to assume a conformation as close as possible to that observed in the cellular prion protein. Furthermore, we have also investigated the peptide interaction with metal cations.

## 2. MATERIAL AND METHODS

### 2.1. Peptide synthesis and characterization

The N- and C-blocked peptides, hPrP[173–195] and hPrP[173–195] D178N, with sequences AcNNFVHDCVNITIKQHTVTTTTKGNH_2_ and AcNNFVHNCVNITIKQHTVTTTTKGNH_2_, respectively, were synthesized by standard fluorenylmethoxycarbonyl chemistry protocol as previously described [29].

### 2.2. Circular dichroism

Far UV CD spectra of both peptides were recorded at room temperature on a Jasco J-810 spectropolarimeter, using 1 cm quartz cell containing 20 *μ*M peptide dissolved in TFE to mimic the *α*-helical structure of the parent segment in the native protein. Spectra were also collected after addition of increasing amounts of metal cations [Zn(II) and Cu(II)] up to a 10 : 1 metal/peptide molar ratio. In any case, final spectra were obtained averaging three scans, subtracting the blank, and converting the signal to mean residue ellipticity in units of deg⋅cm^2^⋅dmol^−1^⋅res^−1^. Other experimental settings were 20 nm/min scan speed, 2.0 nm band width, 0.2 nm resolution, 50 mdeg sensitivity and 4 seconds response.

### 2.3. NMR spectroscopy

All samples were prepared by dissolving the peptide under investigation in TFE_d2_-OH (99%). NMR spectra were acquired at 300 K on a 600 MHz Bruker Avance spectrometer equipped with a cryoprobe. Natural abundance ^1^H-^15^N HSQC and ^1^H-^13^C HSQC [38], TOCSY [39], NOESY, [40] and double quantum filtered COSY [41] spectra were used for resonance assignments. The H_2_O solvent resonance was suppressed using the WATERGATE pulse sequence [42]. NOESY mixing times were set at 200, 300, and 400 milliseconds to follow the NOE buildup rates. TOCSY experiments were recorded with mixing times of 30 and 70 milliseconds. Data were typically apodised with a Gaussian window function and zero-filled to 1 K in f_1_ prior to Fourier transform. NMRPipe [43] and NMRView [44] programs were used for data processing and spectral analysis, respectively. Spin system identification and assignment of individual resonances were carried out by using a combination of TOCSY and DQF-COSY spectra. The TOCSY spectra of all peptides showed well resolved resonances for almost all residues, and sequence specific assignment was obtained by the combined use of TOCSY and NOESY experiments, according to the standard procedure [45]. One-dimensional NMR spectra were also collected after the addition of small aliquots of a 0.5 M ZnCl_2_ aqueous stock solution to the peptide solution.

### 2.4. Structure calculations

NOESY spectra at 300 milliseconds mixing time were used for the integration of NOE cross-peaks. Peak integrals were evaluated by NMRView, transferred to the program package DYANA 1.0.6 [46], and converted to upper distance limits by using the CALIBA [47] module of DYANA. Distance constraints were then worked out by the GRIDSEARCH module to generate a set of allowed dihedral angles. Structure calculation was carried out with the macro ANNEAL module by torsion angle dynamics. Eighty structures were calculated by TSSA, starting with a total of 10000 MD steps and a default value of maximum temperature. The thirty best structures in terms of target functions were considered. A total of 193 and 150 distance restraints were used for structure calculation of hPrP[173–195] and hPrP[173–195] D178N, respectively. These restraints, derived from interresidue, sequential, and medium range NOEs, were introduced in SA torsion space calculation performed by DYANA package. The best thirty structures in terms of root mean square deviation (RMSD) were selected from 80 structures sampled in TSSA calculations.

## 3. RESULTS

### 3.1. hPrP[173–195]

**Figure 1:**
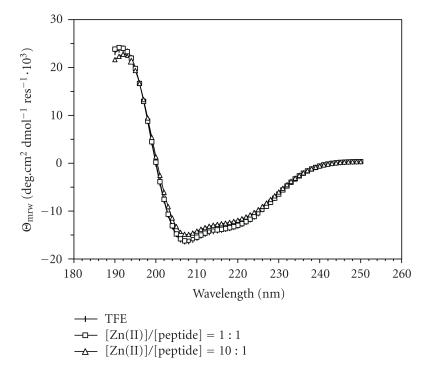
Far UV CD spectra of hPrP[173–195] dissolved in TFE before and after addition of ZnCl_2_ solution. A similar spectral behaviour was observed after titration with CuCl_2_ solution (spectra not shown).

**Figure 2:**
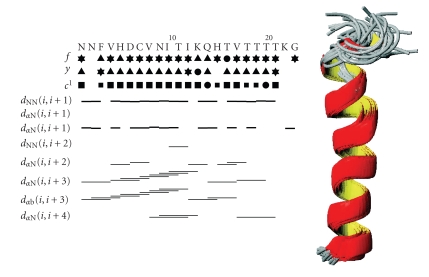
NOE effects and DYANA backbone fitting of hPrP[173–195]. Connectivities were derived from NOESY spectra at 300 milliseconds mixing time. Backbone NOE connectivities are indicated by horizontal lines between residues, with thickness indicating their relative magnitude. The first three lines below the amino acid sequence represent torsion angle restraints for the backbone torsion angles φ and ψ, and for the side-chain torsion angle $\chi_{1}$. For φ and ψ, a 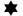 symbol encloses secondary-structure-type conformation; a ▴ symbol indicates compatibility with an ideal *α*-helix; and a • symbol marks a restraint that excludes the torsion angle values of these regular secondary structure elements. Filled squares of different sizes depict torsion angle restraints for $\chi_{1}$, depending on the number of allowed staggered rotamer positions. The bundle of the region 175–193 of the best 30 DYANA structures was obtained by best fitting of the backbone (RMSD = 1.13±0.50 Å).

**Figure 3:**
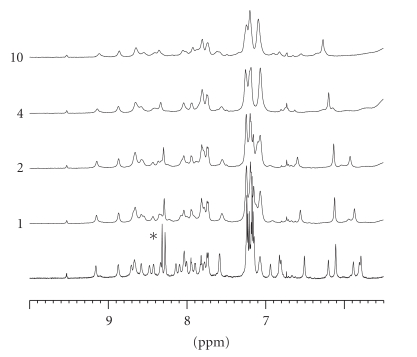
1D NMR spectra of hPrP[173–195] dissolved in TFE-d2 before and after addition of ZnCl_2_ solution. Labels 1, 2, 4, and 10 indicate the total volume (*μ*l) of 0.5 M ZnCl_2_ solution added to 500 *μ*l of 0.6 mM peptide solution, corresponding to Zn(II)/peptide molar ratios of 1.7, 3.3, 6.7, and 16.7, respectively. Imidazolic proton resonances of His residues in metal absence are marked by an asterisk.

As shown in Figure 1, the far UV CD spectrum of hPrP[173–195] in TFE solution shows features typical of *α*-helical conformation. The small spectral alterations that can be noticed on metal titration are likely caused by modification of the dielectric properties of the solvent subsequent to salt addition and do not suggest any specific binding interaction between the peptide and the metal cation. The bar diagram of diagnostic NOE effects, as derived from the NOESY spectrum in TFE at 300 milliseconds mixing time, is reported in Figure 2. Any ambiguity caused by the signals of the four consecutive Thr residues was overcome by NOESY and ^1^H^15^N-HSQC experiments. ^3^ $J_{\text{NH-CH}}$ coupling constants assumed the very small values typical of *α*-helix. Weak $d_{\alpha \text{N(i,i+3)}}$ medium range, strong $d_{\text{NN(i,i+1)}}$ sequential as well as strong $d_{\alpha \beta \text{(i,i+3)}}$ medium range effects for almost all residues are consistent with *α*-helical conformation. Table 1 summarizes torsion angle values and respective order parameters resulting from DYANA calculations. The 175–193 bundle of the best thirty DYANA structures, as obtained by best fitting of the backbone (RMSD = 1.13±0.50 Å), is also drawn in Figure 2. Figure 3 depicts the amidic zone of the 1D spectra after Zn(II) addition. The addition of just one metal ion aliquot was sufficient to cause alteration of the imidazolic proton resonances. Concentration-dependent peptide aggregation on further metal addition caused progressive broadening of all resonances, even causing them to disappear. Overall, this suggests nonspecific metal-peptide interaction, a conclusion that is supported by the unchanged shape of CD spectra, where aggregation did not occur because of the lower peptide concentration.

### 3.2. hPrP[173–195] D178N

**Figure 4:**
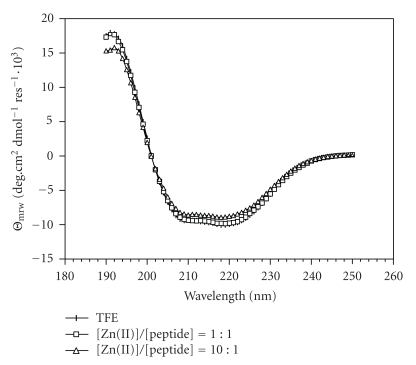
Far UV CD spectra of hPrP[173–195] D178N dissolved in TFE before and after addition of ZnCl_2_ solution. A similar spectral behaviour was observed after titration with CuCl_2_ solution (spectra not shown).

**Figure 5:**
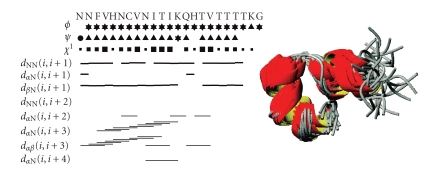
NOE effects and DYANA backbone fitting of hPrP[173–195] D178N. Connectivities were derived from NOESY spectra at 300 milliseconds mixing time. Symbols used for connectivities are the same as reported in Figure 2. The bundle of the region 175–193 of the best 30 DYANA structures was obtained by best fitting of the backbone (RMSD = 2.07±0.61 Å).

**Figure 6:**
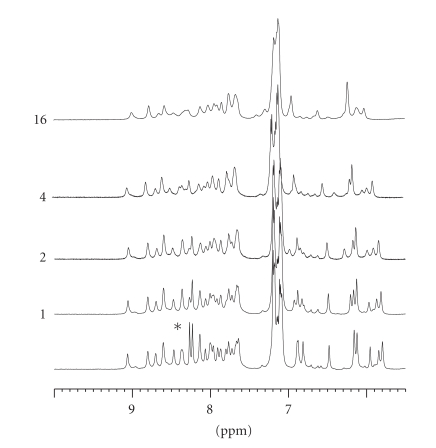
1D NMR spectra of hPrP[173–195] D178N dissolved in TFE-d2 before and after addition of ZnCl_2_ solution. Labels 1, 2, 4, and 16 indicate the total volume (*μ*l) of 0.5 M ZnCl_2_ solution added to 500 *μ*l of 1.0 mM peptide solution, corresponding to Zn(II)/peptide molar ratios of 1.0, 2.0, 4.0, and 16.0, respectively. Imidazolic proton resonances of His residues in metal absence are marked by an asterisk.

The lower intensity of the far UV CD spectrum of hPrP[173–195] D178N, run in the same condition as that of hPrP[173–195] (Figure 4), suggests that the mutant peptide is less helical as compared to the wild type peptide. However, the conclusion that no specific binding interaction with the metal cation can be detected still holds for this peptide. In TOCSY experiments, it has been found that the replacement of Asp178 with Asn does not substantially affect chemical shifts. Only 0.2 ppm protonic chemical shifts of HN and the CH*β* of Asn178 as compared to Asp178 were recorded by superimposition of the two TOCSY experiments. Careful analysis of the NOESY spectrum highlighted that effects typical of secondary structure are essentially located in the N-terminal region, even though the intensity of the $d_{\text{NN(i,i+1)}}$ ones was reduced. The $d_{\alpha \text{N(i,i+1)}}$ between the H-C^*α*^ and the HN-proton of Gln186 and His187, respectively, suggested the local presence of an extended conformation in the modified peptide, strongly perturbing the central core of the wild type helix motif. This is in good agreement with the lower helical content of the CD spectrum of hPrP[173–195] D178N. Figure 5 shows all diagnostic NOE effects as well as the superimposition of the best thirty structures obtained by DYANA calculations (region 175–193). However, the value of the backbone RMSD of 2.07±0.61 Å suggests the presence of several quite similar conformations. Torsion angle values and respective order parameters, as obtained by DYANA calculations (Table 2), validated the presence of a kink centred on Lys185 and Gln186. The amidic zone of 1D NMR spectra of hPrP[173–195] D178N in the presence of various amounts of Zn(II) is reported in Figure 6. The protonic resonances of the His side chains exhibit the same behaviour as that observed for the wild type peptide fragment, but the progressive broadening of side-chain resonances is less relevant. As already observed for hPrP[173–195], these data are not suggestive of well-defined ion-peptide complex formation.

## 4. DISCUSSION

**Figure 7:**
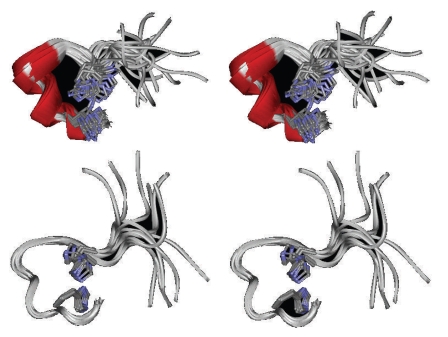
Stereo view of the backbone structure of hPrP[173–195] D178N. Clusters identify two major conformational families, with proximal (top) and distal (bottom) histidine imidazolic rings.

In this work, we report comparative CD and NMR data on the synthetic peptides hPrP[173–195] and hPrP[173–195] D178N, which are related to the *α*2-helical region of the prion protein and represent the wild type sequence and its D178N mutant, respectively, both in the absence and in the presence of metal cations. As can be judged from far UV CD spectra, the two negative bands at 222 and 208 nm, and a positive band at 192 nm indicate that both peptides exhibit *α*-helical arrangement. However, the lower intensity that characterizes the spectrum of the mutant peptide suggests some rearrangement as compared to the single helical structure exhibited by the wild type peptide. In fact, there is NMR evidence that the conformation of the wild type peptide is significantly affected by replacing the negatively charged Asp178 with a neutral Asn residue. In the mutant peptide, increased conformational freedom characterizes all residues downstream Gln188, which ultimately causes unwinding and bending of the wild type fully helical structure. As a consequence, structural rearrangement leads to the formation of two short helices separated by a kink centred on Lys185 and Gln186. In this bent structure, His177 and His187 approach to each other as compared to the parent helical peptide, forming two major conformational families, characterized by proximal and distal imidazole rings, respectively. Moreover, the network of stabilizing H-bonds mainly involves the interaction between Asn174 and Thr188 (head-to-tail type) and between Asn181 and His187 or Gln186 (core type) (Figure 7). In conclusion, we argue that the negative charge of Asp178 plays a key role in forcing the entire 173–195 fragment to assume a full helical conformation.

For both peptides, addition of increasing metal cation aliquots did not perturb NMR spectra in any specific way. The chemical shifts of all resonances did not vary, as it could be expected in case of metal-peptide complex formation, and the overall effect was a progressive generalized broadening of all relevant resonances. In fact, addition of higher and higher metal aliquots caused irreversible aggregation, which always lies in wait when the peptide concentration is very high, possibly owing to ionic strength increase and/or to water addition on metal cation titration. However, that the interaction of the metal with the peptide backbone is nonspecific was confirmed by the unaltered appearance of CD spectra after metal addition, where aggregation did not occur thanks to the lower peptide concentration. These were performed in neat TFE to conform to the conditions of NMR experiments, but further experiments in mixed water/TFE solvent suggested that water-induced effects largely dominate structural rearrangements, rendering metal-induced modifications, if any, hard to discriminate.

Among studies that have been carried out on metal interaction with peptides derived from the PrP C-terminus, it is worth mentioning that recently Brown and coauthors [22] have characterized the formation of different Cu^2+^ complexes in blocked and free C- and N-termini analogues of the peptide fragment 180–193 (VNITKQHTVTTTT), which almost entirely encompasses the ${\text{PrP}}^{\text{C}}$'s *α*2-helix. They suggested that the binding site of copper(II) in the structured region of the protein is located on the His187 residue, and that the anchoring imidazole residue drives the metal coordination environment towards a common binding motif in different regions of the prion protein. Other studies [48] showed that the PrP178–193 peptide has both structural and bioactive properties in common with the amyloidogenic Alzheimer's disease A*β*(25–35) peptide and that the second putative helical region of PrP could be involved in modulation of Cu (II)-mediated toxicity in neurons during prion disease. However, our results suggest that the interaction of metal cations with peptide fragments derived from the C-terminal globular domain could be affected by experimental ambiguity caused by the fact that the structural organization of these peptides is different from that assumed in ${\text{PrP}}^{\text{C}}$. We believe that it is crucial to take this aspect into account when designing experiments aimed at investigating peptide-metal cation interaction.

It is known that the lack of mutual interactions has dramatic effects on the integrity of the whole helical domain of the prion protein, and the stability of one single helical region strongly suffers from ablation of the other helical segments as well as of the disulphide bridge. However, native-like conditions can be to some extent restored choosing a medium that may help extract useful information using the peptide fragment approach. Thus, we have used TFE as the most suitable environment to investigate structural similarities between the wild type and the D178N mutant fragment corresponding to the helix 2. Our experiments confirm that it is reasonable to suspect the involvement of this region in the ${\text{PrP}}^{\text{C}}$-${\text{PrP}}^{\text{Sc}}$ conversion, as emerging evidence points out [34, 49] and we ourselves have suggested elsewhere [29, 50, 51]. As a proof of the structural flexibility of this segment, which can be also inferred from an analysis of PrP pathological variants [52–54], the D178N CJD-associated variant, that is the most important mutation occurring in CJD, is not even able to assume a fully helical structure, like that found in ${\text{PrP}}^{\text{C}}$, in an *α*-inducing environment. This supports the view that the single Asp178 residue is of foremost importance in maintaining the structural properties of the PrP globular domain.

Furthermore, in the peptide fragment approach, it is unlikely that aqueous buffer is the most suitable environment to analyze metal interaction with peptide fragments, whose parent segments in the native protein experience different environmental conditions. Concerning the role played by metal cations in the ${\text{PrP}}^{\text{C}}$-${\text{PrP}}^{\text{Sc}}$ isomerisation, we have shown that the use of the *α*-helix-inducer TFE to force peptides into a conformation close to the helical one that has been found in ${\text{PrP}}^{\text{C}}$ may lead to conclusions different from those that can be obtained studying metal cation interaction with peptides in buffer solution. To embed our results in the body of data on PrP structure and function, it is worth considering that the three-dimensional architecture of ${\text{PrP}}^{\text{C}}$ consists of an unstructured leading tail encompassing residues 23–125 and a C-terminus globular domain, in which residues 126–231 are organized in three *α*-helices and a two-stranded *β*-sheet [55, 56]. Although it is currently believed that the major structural modifications involved in PrP protein misfolding are located in the unstructured N-terminal region, the present work seems to provide further support to evidence accumulated in the literature that the two prion domains play a different role in the prion conversion, stressing that the N-terminal domain is likely the natural target of metal binding; see [57, 58] and references cited therein.

## Figures and Tables

**Table 1:** Torsional angles and order parameters for hPrP[173–195].

Residue	φ	φS	ψ	ψS	$\chi_{1}$	$\chi_{1}$S
Asn^173^	−89.9±4.6	0.997	−54.4±3.2	0.999	−122.6±4.2	0.997
Asn^174^	−59.0±9.9	0.986	−57.2±7.1	0.993	−132.6±86.5	0.253
Phe^175^	−46.0±8.3	0.990	−40.7±13.0	0.976	130.1±53.8	0.626
Val^176^	−69.2±12.9	0.977	−52.4±6.0	0.995	150.2±10.1	0.986
His^177^	−46.7±1.2	1.000	−32.5±4.8	0.997	−156.9±9.4	0.987
Asp^178^	−75.2±2.7	0.999	−41.6±3.8	0.998	−149.7±0.4	1.000
Cys^179^	−64.7±1.0	1.000	−42.8±0.5	1.000	−132.5±0.3	1.000
Val^180^	−47.8±0.6	1.000	−74.0±0.6	1.000	142.5±0.7	1.000
Asn^181^	−49.6±0.3	1.000	−45.9±0.7	1.000	−145.8±0.6	1.000
Ile^182^	−52.6±0.8	1.000	−31.0±1.6	1.000	−74.8±2.8	0.999
Thr^183^	−84.7±3.2	0.999	−38.6±1.4	1.000	−138.2±1.0	1.000
Ile^184^	−73.3±1.7	1.000	−16.2±4.3	0.997	−142.9±3.7	0.998
Lys^185^	−38.0±1.1	1.000	−63.6±0.7	1.000	−159.5±0.5	1.000
Gln^186^	−59.6±9.0	0.988	−46.0±7.8	0.991	−101.4±3.9	0.998
His^187^	−38.9±0.9	1.000	−70.2±7.5	0.992	178.7±38.6	0.796
Thr^188^	−47.8±5.9	0.995	−34.5±2.0	0.999	−99.6±15.2	0.968
Val^189^	−95.1±2.9	0.999	34.2±11.6	0.980	−169.9±9.9	0.986
Thr^190^	−94.6±31.9	0.856	−69.3±15.9	0.963	−48.8±71.3	0.349
Thr^191^	−78.9±46.0	0.724	−15.4±28.1	0.889	−39.8±75.1	0.322
Thr^192^	−75.2±17.2	0.957	−55.5±10.4	0.984	−95.3±37.0	0.818
Thr^193^	−78.4±16.2	0.962	146.4±92.1	0.380	−76.6±92.5	0.139
Lys^194^	45.0±18.0	0.958	94.8±65.5	0.662	−110.9±60.5	0.713
Gly^195^	−16.9±71.7	0.513	49.9±78.6	0.336	—	—

**Table 2:** Torsional angles and order parameters for hPrP[173–195] D178N.

Residue	φ	φS	ψ	ψS	$\chi_{1}$	$\chi_{1}$S
Asn^173^	—	—	−176.7±57.0	0.759	−57.9±52.5	0.673
Asn^174^	−0.4±73.3	0.389	−50.5±10.5	0.984	169.7±83.2	0.294
Phe^175^	−65.1±12.0	0.979	−65.8±14.5	0.969	−114.0±31.8	0.863
Val^176^	−61.1±4.8	0.997	−35.1±15.0	0.967	171.2±7.9	0.991
His^177^	−62.7±8.9	0.988	−13.9±7.2	0.992	−65.7±109.8	0.187
Asn^178^	−58.6±12.6	0.977	−38.1±17.1	0.957	176.4±11.8	0.980
Cys^179^	−75.8±42.6	0.750	−18.4±20.3	0.940	−170.6±18.5	0.950
Val^180^	−102.6±16.5	0.960	−36.5±8.1	0.990	168.1±19.7	0.945
Asn^181^	−57.8±5.7	0.995	−31.0±12.5	0.977	−168.0±12.3	0.978
Ile^182^	−60.2±16.7	0.959	−29.2±19.3	0.947	−71.1±9.6	0.986
Thr^183^	−92.2±25.2	0.910	−34.6±16.0	0.963	−76.3±12.5	0.977
Ile^184^	−82.7±20.7	0.938	−44.3±10.5	0.984	−72.1±7.2	0.992
Lys^185^	79.7±12.8	0.976	2.6±42.2	0.752	−137.8±24.5	0.923
Gln^186^	−142.7±9.7	0.986	−7.6±77.5	0.273	−19.5±86.4	0.208
His^187^	−55.6±50.8	0.687	−111.7±45.2	0.754	−157.1±36.1	0.820
Thr^188^	−23.4±47.8	0.736	−39.8±9.1	0.988	−87.7±36.4	0.816
Val^189^	−54.5±14.3	0.970	−44.6±13.1	0.975	163.8±9.9	0.986
Thr^190^	−48.2±10.2	0.985	−47.6±13.3	0.974	−178.0±70.3	0.516
Thr^191^	−71.1±78.5	0.434	−65.7±13.7	0.973	−38.1±48.4	0.699
Thr^192^	−163.3±70.3	0.455	−51.9±24.0	0.918	−63.3±40.6	0.809
Thr^193^	−103.8±85.0	0.309	−140.6±65.3	0.468	−128.7±42.3	0.751
Lys^194^	89.9±78.6	0.332	129.4±85.7	0.411	−135.6±55.0	0.682
Gly^195^	160.8±70.8	0.444	—	—	—	—
